# Comparative study on free vibration analysis of rotating bi-directional functionally graded beams using multiple beam theories with uncertainty considerations

**DOI:** 10.1038/s41598-023-44411-0

**Published:** 2023-10-20

**Authors:** Moustafa S. Taima, Mohamed B. Shehab, Tamer A. El-Sayed, Michael I. Friswell

**Affiliations:** 1 Department of Mechanical Design, Faculty of Engineering, Mataria, Helwan University, P.O. Box 11718, Helmeiat-Elzaton, Cairo, Egypt; 2 Faculty of Science and Engineering, Swansea University, Bay Campus, Fabian Way, Crymlyn Burrows, Swansea, SA1 8EN UK

**Keywords:** Engineering, Mechanical engineering, Applied mathematics, Composites

## Abstract

The present study investigates the free vibration behavior of rotating beams made of functionally graded materials (FGMs) with a tapered geometry. The material properties of the beams are characterized by an exponential distribution model. The stiffness and mass matrices of the beams are derived using the principle of virtual energy. These matrices are then evaluated using three different beam theories: Bernoulli–Euler (BE) or Classical Beam Theory (CBT), Timoshenko (T) or First-order Shear Deformation Theory (FSDT), and Reddy (R) or Third-order Shear Deformation Theory (TSDT). Additionally, the study incorporates uncertainties in the model parameters, including rotational velocity, beam material properties, and material distribution. The mean-centered second-order perturbation method is employed to account for the randomness of these properties. To ensure the robustness and accuracy of the probabilistic framework, numerical examples are presented, and the results are compared with those obtained through the Monte Carlo simulation technique. The investigation explores the impact of critical parameters, including material distribution, taper ratios, aspect ratio, hub radius, and rotational speed, on the natural frequencies of the beams is explored within the scope of this investigation. The outcomes are compared not only with previously published research findings but also with the results of 3-Dimensional Finite Element (3D-FE) simulations conducted using ANSYS to validate the model’s effectiveness. The comparisons demonstrate a strong agreement across all evaluations. Specifically, it is observed that for thick beams, the results obtained from FSDT and TSDT exhibit a greater agreement with the 3D-FE simulations compared to CBT. It is shown that the coefficient of variation (C.O.V.) of first mode eigenvalue of TSDT, FSDT and CBT are approximately identical for random rotational velocity and discernible deviations are noted in CBT compared to FSDT and TSDT in the case of random material properties. The findings suggest that TSDT outperforms FSDT by eliminating the need for a shear correction coefficient, thereby establishing its superiority in accurately predicting the natural frequencies of rotating, tapered beams composed of FGMs.

## Introduction

Rotating beams can be used to simulate structures such as industrial fans, helicopter and propeller blades, wind and steam turbines, robot manipulators, and spinning space structures^1^. Understanding the dynamical behavior of rotating beams is critical during the early design stages to avoid resonance within the operational speed range and to evaluate rotating beam performance. These structural components are made of various material classes to satisfy various engineering design specifications. Because of their superior properties, rotating beams manufactured from composite materials have been widely applied in a range of industrial applications during the last few decades. When compared to traditional engineering materials, the advantages of composite materials, together with their ability to customize their designs to specific uses, have given them a competitive advantage. Aksencer and Aydogdu^2^ investigated the free vibration of a rotating laminated composite beam with attached mass using the Ritz method. The authors used different beam theories (CBT, FSDT, and TSDT) in the formulation and considered the cross-ply lamination configuration. In addition, attached mass to beam mass ratio, position of the mass and aspect ratio were examined. Based on Timoshenko beam theory and nonlinear strain-displacement, Khosravi et al.^3^ discussed the thermal vibration of rotating composite beams. The beams were reinforced by Carbon Nano-Tubes (CNTs) according to a uniform and two symmetric gradient distributions. The authors used the differential transform method to obtain the natural frequencies for different parameters such as hub radius, rotating speed, aspect ratio, temperature, and boundary conditions. Xu et al.^4^ used the TSDT with the Ritz technique to investigate the first resonance frequency of rotating nanocomposite beams reinforced with carbon nanotubes. Mohammadi et al.^5^ used the Differential Quadrature Method (DQM) to solve the governing equations for a rotating beam composed of multilayer piezoelectric nanobeams. The authors employed surface elasticity theory in conjunction with nonlocal continuum theory for the Timoshenko beam to derive the equations of motion. Furthermore, they investigated the effects of four different boundary conditions, thermal stress, external voltage, and angular velocity on the natural frequency.

A class of composites called Functionally Graded Materials (FGMs) has attracted significant attention in several modern engineering applications. The required variational features of the material combinations are increased by the application of FGMs to increase functional performance. Because of this continual fluctuation, FGMs have a continuous stress distribution and prevent stress concentrations. Another exceptional quality of FGMs is their capacity to endure high temperatures while preserving structural integrity. Akbaş^6^ used Lagrange’s equations to derive the equations of motions and then the Finite Element Method (FEM) to obtain the thermal natural frequencies for axially FGM Bernoulli-Euler beam. The material properties were changed by a power-law and were temperature dependent. To examine the nonlinear free thermal vibration of pre/post buckled rotating FGM beams, Arvin et al.^7^ employed Bernoulli-Euler theory and a nonlinear strain displacement relationship to propose some new algorithms in conjunction with the nonlinear finite element method. Van Dang^8^ applied Bernoulli-Euler theory and the FEM to investigate the static bending of functionally graded porous rotating beams. The beam was impacted by lateral and axial compressive force and embedded in an elastic foundation with two parameters. The material properties vary in the thickness direction according to the power law. Binh et al.^9^ obtained and solved the equations of motion for a rotating Timoshenko beam formed of functionally graded porous material reinforced by graphene platelets using the Chebyshev–Ritz method. The material characteristics change according to two different types of porosity distributions through thickness and two different graphene platelet dispersion patterns. The authors investigated the influence of rotational speed, hub radius, porosity, and weight fraction on the natural frequency. Dang et al.^10^ considered the Coriolis and centrifugal forces in deriving the equations of motion by using Hamilton’s principle with Love’s shell theory for a cylindrical shell made of Functionally Graded Porous (FGP) material. The porosity was changed through the thickness according to three different porosity distributions. The authors used Galerkin’s method to obtain the natural frequencies for different boundary conditions. Also, they studied the effect of porosity distribution, rotational speed, Coriolis acceleration, and geometric parameters.

Unfortunately, the 1D-FGMs are ineffective at meeting the technical specifications for shuttles and aerospace craft, such as the stress distributions and temperature in various directions^11^. Utilizing material properties that change in desirable directions, such as two-directional FGM, can address this limitation^12^. Fang et al.^13^ investigated the time response and coupled axial, flap-wise, and chord-wise vibration of a rotating BFGM cantilever beam. The beam material was gradually changed according to a power-law though the width and thickness. Lagrange’s equation and the Ritz method were used to derive the dynamic equations and then solved by using the state space method for different material gradients and rotating speeds.

Rotating beams can be classified based on their geometric properties as either uniform or tapered. The later is often preferred due to its ability to provide an optimal distribution of weight and strength, which is particularly useful in meeting specific structural and functional requirements^14^. Banerjee et al.^15^ used Hamilton’s principle to derive the equations of motion for the flap vibration of a rotating double tapered Bernoulli-Euler beam. The author used the Wittrick-Williams algorithm to solve the resulting dynamic stiffness matrix for different taper ratios, rotational speeds, and hub radii. Lagrange’s form with the FEM were used to develop mass, elastic, and centrifugal stiffness matrices for a rotating tapered Bernoulli-Euler beam by Bazoune^14^. The author examined the tapering effect in two planes, hub radius, and rotational speed. Chen et al.^16^ examined the accuracy and efficiency of the variational iteration method for the free vibration analysis of a rotating Timoshenko beam. The beam was linearly tapered though the width and height. Adair and Jaeger^17^ reformulated the fourth-order differential equation as a first order matrix and used the power series method to obtain the natural frequencies of a rotating taper Bernoulli-Euler beam. The authors studied both cone and wedge cantilever beams for different taper ratios. Nourifar et al.^18^ compared the differential transform method and the finite element method for the vibration of a rotating cylindrical tapered Bernoulli-Euler beam. The effect of rotating speed and taper ratio were examined on the natural frequencies. An improved transfer matrix method was developed by Lee and Lee^19^ to obtain the bending natural frequencies for a tapered rotating Bernoulli-Euler beam. The Frobenius method for a power series was used to solve the equations of motion. The authors studied the effect of centrifugal axial force, taper ratio, and hub radius on the natural frequencies. Wang and Li^20^ used the differential quadrature method to solve the differential equations obtained by Hamilton’s principle for the lateral vibration of a tapered rotating hollow beam. The beam has constant inner cross-section radius and tapered outer radius. The authors examined the effect of rotating speed, hub radius, aspect ratio, taper ratio, and inner radius.

Some literature has explored the use of both taper and composite materials in rotating beams. Piovan and Sampaio^21^ used the variational principle to develop a nonlinear model for a rotating FGM tapered Timoshenko beam. The FEM was used to obtain the natural frequencies for different material distribution, aspect ratios, and speeds. Zarrinzadeh et al.^22^ conducted an in-depth investigation of the vibration characteristics of a rotating tapered axially functionally graded material (AFGM) Bernoulli-Euler beam using the finite element method. Their study encompassed a systematic exploration of various influential parameters, including material properties, taper ratio, rotational speed, hub radius, boundary conditions, and the presence of a tip mass. For both Bernoulli-Euler and Timoshenko rotating tapered FGM beams, Hajheidaria et al.^23^ investigated the lead-lag, flap, and flap-lag vibration. The metal/ceramic-based FGM beam properties changed though the thickness according to a power-law in a symmetric structure. The authors used the finite element method with 4 DOF and 8 DOF element models. Also, they studied the effect of volume fraction, rotational speed, hub radius, and taper ratio. Kumar et al.^24^ used the differential transform method to estimate the flap wise natural frequencies of tapered FGM beams. The material properties changed laterally from the middle to the outer surface symmetrically and were estimated using Mori Tanaka methods. The authors discussed the effect of rotational speed, hub radius, taper ratio, and gradient index on the frequency.

The modified variational method and multidomain mixed approximations were used by Tian et al.^25^ to investigate the vibration analysis of a double-tapered rotating FGM beam. The beam material including porosities was distributed based on the modified rule of mixtures. In this model, the Coriolis and nonlinear effects were considered for bending-stretching, twist-stretching, and bending twist vibration modes. The authors investigated the material, rotation speed, and various geometric effects. Bhattacharya and Das^26^ considered the non-linear geometry, Coriolis acceleration, spin-softening, and thermal environment to study the free vibration of rotating micro-beams. In this study, Timoshenko theory, along with modified couple stress theory, investigated a double taper BFGM rotating beam. Also, the authors examined the effect of FGM composition, size-dependence, taper ratio, aspect ratio, hub radius, and temperature. To obtain the natural frequencies and mode shapes of a rotating BFGM for a tapered cantilever beam, Zhou et al.^27^ used the Rayleigh-Ritz method. The equations of motion for time-dependent rotating velocity with periodic coefficients were derived by using Hamilton’s principle and the Galerkin method. Bolotin’s method with a higher-order approximation is used to solve the dynamic instability caused by periodic rotational velocity. The authors examined the effect of hub radius, rotational speed, FGM index, dynamic amplitude factor, and taper ratio on dynamic instability and natural frequency. Özdemir^28^ investigated the free vibration and buckling behavior of rotating beams. Considerations included linearly tapered beams and axially functionally graded materials using a simple power law. Bernoulli-Euler and Timoshenko beam theories were applied using the Finite Element Method. The author investigated many parameters including the hub radius, rotational speed, power law index, aspect ratio, taper ratio, and other boundary conditions.

Various beam theories have been employed to investigate the vibration characteristics of rotating beams. The classical beam theory, also known as Bernoulli-Euler theory (CBT), represents the oldest and most fundamental approach. CBT assumes that the cross-section of the beam remains planar and perpendicular to the beam axis after deformation. Due to its simplicity and suitability for thin beams where transverse shear deformation is less significant, CBT continues to be widely utilized^6–8,14,15,17–20,22–24,27,29^.

For situations involving thick beams, Timoshenko beam theory, categorized as a First-order Shear Deformation Theory (FSDT), is commonly employed^3,5,9,16,21,23,26,30–39^. In FSDT, the assumption of the cross-section staying perpendicular to the beam axis after deformation is no longer taken for granted. Additionally, the shear distribution across the beam section is approximated as constant. To compensate for the uniform shear distribution, a shear correction factor $\kappa _s$ is introduced in this theory.

Third-order shear deformation theory (TSDT), also known as Reddy beam theory, goes a step further, and accounts for the fact that the cross-section will no longer remains straight or perpendicular to the beam axis after deformation. In TSDT, the transverse shear strain and stress are assumed to have a parabolic distribution with respect to the thickness coordinate^4,8,40^.

In the literature, there is a paucity of studies that compare different beam theories for rotating beams. However, Hajheidaria et al.^23^ and Özdemir^28^ discussed and compared Bernoulli-Euler and Timoshenko beam theories. Furthermore, Aksencer and Aydogdu^2^ conducted a comparison of the Reddy, Timoshenko, and Bernoulli-Euler beam theories for rotating beams. These studies provide important insights into the performance of different beam theories and can assist in the design and analysis of rotating beam structures. Nonetheless, further research is needed to fully understand the behavior of rotating beams and determine the most appropriate beam theory for specific applications.

The prior investigations have primarily focused on the vibration of rotating beams characterized by deterministic properties. Nevertheless, it is crucial to acknowledge that real-world structures and mechanical systems inherently possess random properties. These uncertainties have a significant impact on both performance and structural reliability. Within the realm of rotating structural systems, these uncertainties arise from various sources, including variations in loads and material properties. To design highly reliable rotating beam structures, it is essential to comprehensively examine the collective effects of uncertainties in material and sectional properties, geometric parameters, and angular velocity on the stochastic response of rotating beams. Furthermore, conducting sensitivity analyses is crucial as it allows us to identify the design parameters that have the most significant influence on the computed statistical characteristics of the structural responses. Several numerical studies have been conducted to explore uncertainties in the dynamics of rotating beams^41–44^. However, based on the existing literature and the authors’ knowledge, no study has explored the uncertainty associated with material distribution in functionally graded material (FGM) rotating beams and its impact on their natural frequencies.

The vibration analysis of rotating beams is crucial for various engineering applications, and it is necessary to identify the appropriate beam theory for specific scenarios. However, there is a lack of studies in the literature that compare different beam theories for rotating beams with BFGM and tapered geometries. This study aims to address this research gap by comparing the performance of three different beam theories: Bernoulli–Euler, Timoshenko, and Reddy, using finite element analysis with three-dimensional finite elements using the ANSYS software. Thus, this study is significant as it will provide valuable insights into the behavior of rotating FGM tapered beams and determine the most appropriate beam theory for specific applications. Additionally, uncertainties are addressed by incorporating them into the analysis of rotating velocity, beam material, and material distribution. Key parameters, including elasticity modulus, shear modulus, density, and material distribution, are treated as random fields, while rotational speed is regarded as a random variable. The remaining sections of this article are organized as follows: Section “Mathematical model formulation” presents the theoretical model based on the three theories and the finite element analysis using the ANSYS software. In Section “Results and discussion”, examples of verification are showcased, and the corresponding model results are presented. Finally, the conclusions section summarizes the key findings of this work, highlighting its contributions to the existing literature and potential advancements in the field.

## Mathematical model formulation

**Figure 1:**
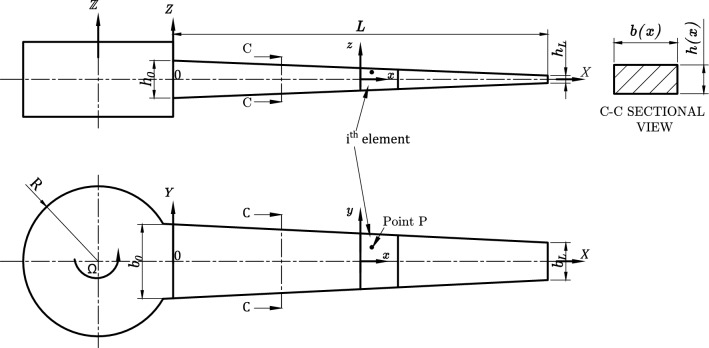
Schematic drawing of a double tapered rotating BFGM beam.

A bi-directional functionally graded material beam of total length *L* along the axial axis *X* and a double tapered cross-sectional area $A\left( X\right)$ is shown in Fig. 1. $b\left( X\right)$ is the width and parallel to the *Y* axis and $h\left( X\right)$ is the thickness and parallel to the *Z* axis. The beam is attached to a rigid hub of radius *R*. The beam rotates in the $X-Y$ plane with a constant angular speed $\Omega$ in rad/s about the ${\mathbb {Z}}$ axis. Also, the beam is divided into $N_e$ elements with equal length $\ell$ and has a local coordinate system $x,\ y,\ z$, where $X,\ Y,\ Z$ is the global coordinate system. $L_i$ denotes the offset of the *i*th element from the *Z*-axis as follows:

1 $$\begin{aligned} L_i=\left( i-1\right) {\frac{L}{N_e} }\end{aligned}$$

### Geometry and material properties

The double tapered beam geometrical dimensions and cross-sectional area and moment of inertia is given as a function of *x* by

2 $$\begin{aligned} b(x)&= b_0\left( 1-C_b\frac{x+L_i}{L}\right) \nonumber \\ h(x)&= h_0\left( 1-C_h\frac{x+L_i}{L}\right) \nonumber \\ A(x)&= A_0\left( 1-C_b\frac{x+L_i}{L}\right) \left( 1-C_h\frac{x+L_i}{L}\right) \nonumber \\ I_{yy}(x)&= I_{yy_0}\left( 1-C_b\frac{x+L_i}{L}\right) \left( 1-C_h\frac{x+L_i}{L}\right) ^3 \end{aligned}$$

where $x+L_i=X$ and $h_0$, $b_0$, $A_0$ and $I_{yy_0}$ are the thickness, width, cross-sectional area and second area moment of inertia at the beam root, respectively. $h_L$ and $b_L$ denote the beam thickness and width at the free end. Also, $0\le C_b=1-\frac{b_L}{b_0}<1$ is the width taper ratio and $0\le C_h=1-\frac{h_L}{h_0}<1$ is the thickness taper ratio. It is noted that $C_b=C_h=0$ for a uniform beam cross section and for $C_b\ne C_h$ the beam has different taper values in the width and thickness directions.

In this work, the beam material properties vary continuously along the beam thickness or the longitudinal direction or both according to exponential rule of mixtures^12^. Thus

3 $$\begin{aligned} P\left( x,z\right) =P_0e^{g_x\left( \frac{x+L_i}{L}\right) +g_z\left( \frac{z}{h\left( x\right) }+\frac{1}{2}\right) }, \end{aligned}$$

where $P\left( x,z\right)$ is the effective material properties (Young’s modulus *E*, density $\rho$ and shear modulus *G*) and $P_0$ is the material property at the reference position $\left( 0,-\frac{h_0}{2}\right)$. $g_x$ and $g_z$ are the gradient indexes through the longitudinal and thickness direction, respectively. The beam material is homogeneous for $g_x=g_z=0$.

### Displacement and strain fields

**Figure 2:**
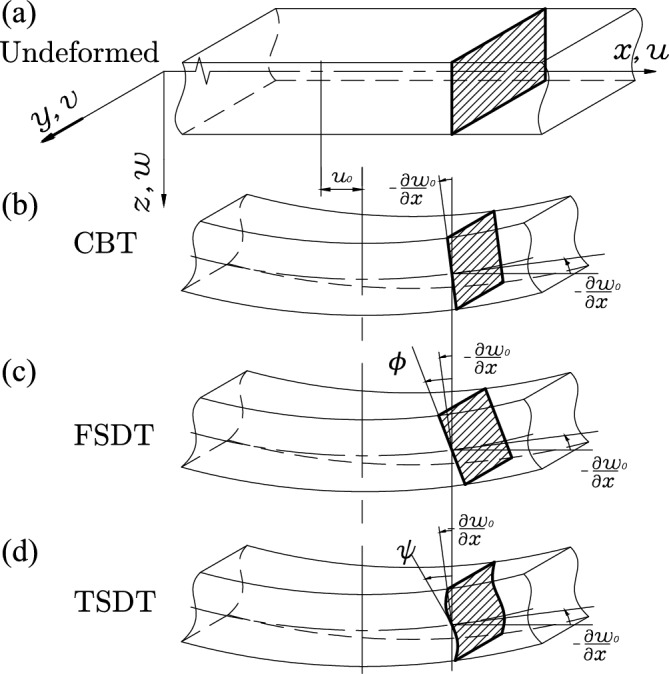
The cross-section deformation for CBT, FSDT, and TSDT theories^45^.

In the current work, the coupled axial and flap-wise transverse vibration is considered; hence the lag-wise or twisting vibration is not considered. The axial displacement *u* and transverse displacement *w* of any point on the beam according to CBT, FSDT, and TSDT are given by Eqs. (4)–(6), respectively and shown in Fig. 2b–d respectively. Figure 2a represents the undeformed cross-section for reference.

4 $$\begin{aligned} \begin{aligned} u^C\left( x,z,t\right)&= u_0-z\frac{\partial w_0}{\partial x} \\ w^C\left( x,z,t\right)&= w_0 \end{aligned} \end{aligned}$$

5 $$\begin{aligned} &u^F\left( x,z,t\right) =u_0-z\phi \\&w^F\left( x,z,t\right) =w_0 \end{aligned}$$

6 $$\begin{aligned} &u^T\left( x,z,t\right) =u_0+z\psi -\frac{4}{3h^2}z^3\left( \psi +\frac{\partial w_0}{\partial x}\right) \\&w^T\left( x,z,t\right) =w_0 \end{aligned}$$

The superscripts $\left( \ \right) ^C$, $\left( \ \right) ^F$, and $\left( \ \right) ^T$ are used to represent CBT, FSDT, and TSDT, respectively. $u_0$ and $w_0$ are the axial and transverse deflection on the neutral axis (i.e., $z=0$) and *t* is time. $\frac{\partial w_0}{\partial x}$ denotes the pure bending cross-section slope in CBT, $\phi$ is the cross-section rotation in FSDT and $\psi$ is the slope of the deformed line at $z=0$ in TSDT as shown in Fig. 2b–d respectively.

Equations (4)–(6) can be rewritten in terms of the displacement vector $\mathbf {{d}_{s}}$ as:

7 $$\begin{aligned} \left\{ d_s\right\} ^C=\left\{ \begin{matrix} u\\ w\\ \end{matrix} \right\} = \left[ \begin{matrix} 1&{}0&{}-z\\ 0&{}1&{}0\\ \end{matrix} \right] \left\{ \begin{matrix} u_0 \ w_0 \ w_0^\prime \end{matrix} \right\} ^\intercal \end{aligned}$$

8 $$\begin{aligned} \left\{ d_s\right\} ^F=\left\{ \begin{matrix} u\\ w\\ \end{matrix} \right\} =\left[ \begin{matrix} 1&{}0&{}-z\\ 0&{}1&{}0\\ \end{matrix} \right] \left\{ \begin{matrix} u_0 \ w_0 \ \phi \end{matrix} \right\} ^\intercal \end{aligned}$$

9 $$\begin{aligned} \left\{ d_s\right\} ^T= \left\{ \begin{matrix} u\\ w\\ \end{matrix} \right\} = \left[ \begin{matrix} 1&{}0&{}-\frac{4}{3h^2}z^3&{}z-\frac{4}{3h^2}z^3\\ 0&{}1&{}0&{}0\\ \end{matrix} \right] \left\{ \begin{matrix} u_0 \ w_0 \ w_0^\prime \ \psi \end{matrix} \right\} ^\intercal \end{aligned}$$

where the superscript prime $\left( \ \ \right) ^\prime$ denotes the derivative with respect to *x* and the superscript $^\intercal$ denotes the transpose. For linear and small deformations, the non-zero strain fields can be represented by Eqs. (10)–(12) for CBT, FSDT, and TSDT, respectively.

10 $$\begin{aligned} \varepsilon _{xx}^C = \frac{\partial u}{\partial x} = u_0^\prime - zw_0^{\prime \prime } \end{aligned}$$

11 $$\begin{aligned} &\varepsilon _{xx}^F = \frac{\partial u}{\partial x} = u_0^\prime - z\phi ^\prime \quad \\&\gamma _{xz}^F = -\phi + w_0^\prime \end{aligned}$$

12 $$\begin{aligned}&\varepsilon _{xx}^T = \frac{\partial u}{\partial x} = u_0^\prime - \frac{4}{3h^2}z^3w_0^{\prime \prime } + \left( z - \frac{4}{3h^2}z^3\right) \psi ^\prime \quad \\&\gamma _{xz}^T = \left( 1-\frac{4}{h^2}z^2\right) \left( w_0^\prime + \psi \right) \end{aligned}$$

or in vector form as

13 $$\begin{aligned} {\varepsilon }^C= \left[ \begin{matrix} 1&{}-z\\ \end{matrix} \right] \left\{ \begin{matrix} u_0^\prime \ w_0^{^{\prime \prime }\hspace{-0.66666pt}} \end{matrix} \right\} ^\intercal \end{aligned}$$

14 $$\begin{aligned} {\varepsilon }^F=\left\{ \begin{matrix} \varepsilon _{xx}\\ \gamma _{xz}\\ \end{matrix}\right\} = \left[ \begin{matrix} 1&{}0&{}0&{}-z\\ 0&{}1&{}-1&{}0\\ \end{matrix}\right] \left\{ \begin{matrix} u_0^\prime \ w_0^\prime \ \phi \ \phi ^\prime \end{matrix} \right\} ^\intercal \end{aligned}$$

15 $$\begin{aligned} {\varepsilon }^T=\left\{ \begin{matrix} \varepsilon _{xx}\\ \gamma _{xz}\\ \end{matrix}\right\} = \left[ \begin{matrix} 1&{}0&{}-\frac{4}{3h^2}z^3&{}0&{}z-\frac{4}{3h^2}z^3\\ 0&{}1-\frac{4}{h^2}z^2&{}0&{}1-\frac{4}{h^2}z^2&{}0\\ \end{matrix}\right] \left\{ \begin{matrix} u_0^\prime \ w_0^\prime \ w_0^{^{\prime \prime }\hspace{-0.66666pt}} \ \psi \ \psi ^\prime \end{matrix}\right\} ^\intercal \end{aligned}$$

### Stress–strain constitutive equations

The stress–strain constitutive equations according to Hooke’s law for linear and small deformations can be considered for the FGM beam for CBT, FSDT, and TSDT beam theory as, respectively:

16 $$\begin{aligned} \sigma ^C=\sigma _{xx}=E\left( x,y,z\right) \varepsilon _{xx} \end{aligned}$$

17 $$\begin{aligned} \sigma ^F=\left\{ \begin{matrix} \sigma _{xx}\\ \tau _{xz}\\ \end{matrix}\right\} = \left[ \begin{matrix} E\left( x,y,z\right) &{}0\\ 0&{}\kappa _sG\left( x,y,z\right) \\ \end{matrix}\right] \left\{ \begin{matrix} \varepsilon _{xx}\\ \gamma _{xz}\\ \end{matrix}\right\} \end{aligned}$$

18 $$\begin{aligned} \sigma ^T=\left\{ \begin{matrix} \sigma _{xx}\\ \tau _{xz}\\ \end{matrix}\right\} = \left[ \begin{matrix} E\left( x,y,z\right) &{}0\\ 0&{}G\left( x,y,z\right) \\ \end{matrix}\right] \left\{ \begin{matrix} \varepsilon _{xx}\\ \gamma _{xz}\\ \end{matrix}\right\} \end{aligned}$$

where $\kappa _s$ denotes the shear correction factor for FSDT.

### Virtual energy expressions

The virtual potential energy expression due to the stress field of a single beam element can be obtained in the form

19 $$\begin{aligned} \delta \text{PE}_s=\iiint _{{\mathbb {V}}}{\sigma ^\intercal \delta \varepsilon }\text{d}{\mathbb {V}} \end{aligned}$$

where the subscript *s* indicates stress field, the superscript $^\intercal$ denotes the transpose and ${\mathbb {V}}$ is the volume. Substituting Eqs. (13)–(18) into Eq. (19) gives the virtual strain energy for CBT, FSDT, and TSDT, respectively, as

20 $$\begin{aligned} {\delta PE}_s^C = \int _{0}^{\ell } \left[ \begin{Bmatrix} u_0^\prime \ w_0^{\prime \prime } \end{Bmatrix} \ \ {\textbf{D}}^C \ \ \delta \begin{Bmatrix} u_0^\prime \ w_0^{\prime \prime } \end{Bmatrix}^\intercal \,\right] \text{d}x \end{aligned}$$

21 $$\begin{aligned} {\delta PE}_s^F = \int _{0}^{\ell } \left[ \begin{Bmatrix} u_0^\prime \ w_0^\prime \ \phi \ \phi ^\prime \end{Bmatrix} \ \ {\textbf{D}}^F \ \delta \begin{Bmatrix} u_0^\prime \ w_0^\prime \ \phi \ \phi ^\prime \end{Bmatrix}^\intercal \right] \, \text{d}x \end{aligned}$$

22 $$\begin{aligned} {\delta PE}_s^T = \int _{0}^{\ell } \left[ \begin{Bmatrix} u_0^\prime \ w_0^\prime \ w_0^{\prime \prime } \ \psi \ \psi ^\prime \end{Bmatrix} \ \ {\textbf{D}}^T \ \ \delta \begin{Bmatrix} u_0^\prime \ w_0^\prime \ w_0^{\prime \prime } \ \psi \ \psi ^\prime \end{Bmatrix}^\intercal \right] \, \text{d}x \end{aligned}$$

where $$D^C=\int _{-\frac{h(x)}{2}}^{\frac{h(x)}{2}}{\int _{-\frac{b(x)}{2}}^{\frac{b(x)}{2}}{[\mathbf {\sigma }^C \ \ \ \mathbf {\varepsilon }^C]}\,\text{d}y\,\text{d}z}$$, $$D^F=\int _{-\frac{h(x)}{2}}^{\frac{h(x)}{2}}{\int _{-\frac{b(x)}{2}}^{\frac{b(x)}{2}}{[\mathbf {\sigma }^F\ \ \ \mathbf {\varepsilon }^F]}\,\text{d}y\,\text{d}z}$$, and $$D^T=\int _{-\frac{h(x)}{2}}^{\frac{h(x)}{2}}{\int _{-\frac{b(x)}{2}}^{\frac{b(x)}{2}}{[\mathbf {\sigma }^T\ \ \ \mathbf {\varepsilon }^T]}\,\text{d}y\,\text{d}z}$$.

The virtual potential energy expression due to the axial centrifugal force of a single beam element is given by

23 $$\begin{aligned} \delta \text{PE}_{cf} = \int _{0}^{\ell } {F_{cf}w_0^\prime \delta w_0^\prime } \text{d}x \quad , \end{aligned}$$

where the subscript *cf* denotes centrifugal, and $F_{cf}$ is the centrifugal force that can be obtained as

24 $$\begin{aligned} F_{cf}(x) = \int _{x}^{L} \int _{-\frac{h(x)}{2}}^{\frac{h(x)}{2}} \int _{-\frac{b(x)}{2}}^{\frac{b(x)}{2}} \rho (x,z)\Omega ^2 \left( x+L_i\right) \text{d}y \, \text{d}z \, \text{d}x \quad \end{aligned}$$

The virtual kinetic energy expression of a single beam element is

25 $$\begin{aligned} \delta KE = \iiint _{{\mathbb {V}}} \rho \left\{ \text{d}{\dot{s}}\right\} ^\intercal \delta \left\{ \text{d}{\dot{s}}\right\} \text{d}{\mathbb {V}}, \quad \end{aligned}$$

where the superscript dot $\dot{\left( \ \ \right) }$ indicates the time derivative. The specific virtual kinetic energy for CBT, FSDT, and TSDT can be obtained by substituting Eqs. (7)–(9) into the general form of virtual kinetic energy Eq. (25) as:

26 $$\begin{aligned} \delta {KE}^C = \int _{0}^{\ell } \left[ \begin{Bmatrix} \dot{u_0} \ \dot{w_0} \ {\dot{w_0}}^\prime \end{Bmatrix} \ \ {\textbf{H}}^C \ \ \ \delta \begin{Bmatrix} \dot{u_0} \ \dot{w_0} \ {\dot{w_0}}^\prime \end{Bmatrix}^\intercal \right] \text{d}x \quad \end{aligned}$$

27 $$\begin{aligned} \delta {KE}^F = \int _{0}^{\ell } \left[ \begin{Bmatrix} \dot{u_0} \ \dot{w_0} \ {\dot{\phi }} \end{Bmatrix} \ \ {\textbf{H}}^F \ \ \delta \begin{Bmatrix} \dot{u_0} \ \dot{w_0} \ {\dot{\phi }} \end{Bmatrix}^\intercal \right] \text{d}x \quad \end{aligned}$$

28 $$\begin{aligned} \delta {KE}^T = \int _{0}^{\ell } \left[ \begin{Bmatrix} \dot{u_0} \ \dot{w_0} \ \dot{w_0^\prime } \ {\dot{\psi }} \end{Bmatrix} \ \ {\textbf{H}}^T \ \delta \begin{Bmatrix} \dot{u_0} \ \dot{w_0} \ \dot{w_0^\prime } \ {\dot{\psi }} \end{Bmatrix}^\intercal \right] \text{d}x \quad \end{aligned}$$

where $$H^C=\int _{-\frac{h(x)}{2}}^{\frac{h(x)}{2}}{\int _{-\frac{b(x)}{2}}^{\frac{b(x)}{2}}{ [\rho \{d_s\}^C \ \ \varvec{\delta }\{d_s\}^C] }\,\text{d}y\,\text{d}z}$$, $$H^F=\int _{-\frac{h(x)}{2}}^{\frac{h(x)}{2}}{\int _{-\frac{b(x)}{2}}^{\frac{b(x)}{2}}{ [ \rho \{d_s\}^F \ \ \varvec{\delta }\{d_s\}^F}\,] \ }\text{d}y\,\text{d}z$$, and $$H^T=\int _{-\frac{h(x)}{2}}^{\frac{h(x)}{2}}{\int _{-\frac{b(x)}{2}}^{\frac{b(x)}{2}}{ [ \rho \{d_s\}^T \ \ \varvec{\delta }\{d_s\}^T] }\,\text{d}y\,\text{d}z}$$.

### Finite element modeling

The condition of equilibrium of a dynamical structure for free vibration based on the principle of virtual energy is

29 $$\begin{aligned} \delta PE_s+\delta PE_{cf}-\delta KE=0. \end{aligned}$$

**Figure 3:**
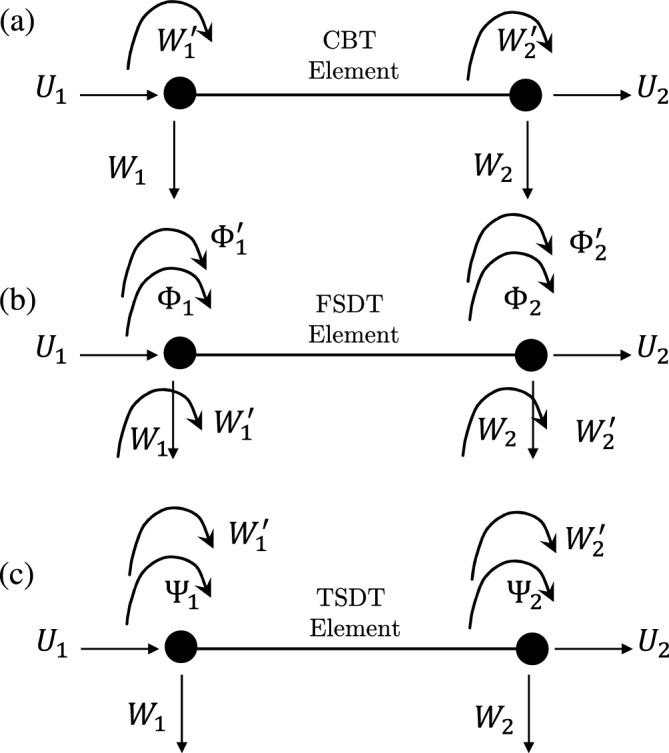
The beam elements for CBT, FSDT, and TSDT theories.

To accurately analyze the vibration behavior of rotating double taper beams made of BFGM, FEM techniques are employed using three distinct theories: CBT, FSDT, and TSDT. Each theory requires a specific number of degrees of freedom (DOFs) to adequately capture the axial and transverse displacements, as well as the rotational motion of the beam. In the CBT approach, a two-node, six DOFs element is utilized to capture the desired displacement and rotation components. These DOFs comprise two DOFs for axial displacement, two for transverse displacement, and an additional two for rotational bending motion, as illustrated in Fig. 3a. Axial displacement is estimated using Lagrange linear shape functions, while the transverse displacement is estimated using Hermitian shape functions as given in Eq. (30).

To achieve higher precision in the analysis, the FSDT method is employed, utilizing a two-node, ten DOFs element to accurately capture axial and transverse displacements, as well as rotation. The ten DOFs element for Timoshenko beam analysis offers advantages over the six-DOF element, including a superior convergence rate^23^. The nodal displacement vector in this model consists of two degrees of freedom for axial displacement, two for transverse displacement, two for rotational motion, and their corresponding derivatives as shown in Fig. 3b. Axial displacement is estimated using Lagrange linear shape functions, while transverse displacement and rotation are estimated using Hermitian shape functions based on the nodal displacements as given in Eq. (31).

For enhanced accuracy in the analysis, TSDT theory is utilized, employing a two-node, eight DOFs element. These eight DOFs encompass two degrees of freedom for axial displacement, two for transverse displacement, two for the derivative of transverse displacement, and two for rotation as shown in Fig. 3c. The estimation of axial displacement and rotation relies on Lagrange linear shape functions, while transverse displacement is approximated using Hermitian shape functions, both formulated in terms of nodal displacements as given in Eq. (32).

30 $$\begin{aligned} \{q^C\}&= [N^C]\{q_e^C\} \end{aligned}$$

31 $$\begin{aligned} \{q^F\}&= [N^F]\{q_e^F\} \end{aligned}$$

32 $$\begin{aligned} \{q^T\}&= [N^T]\{q_e^T\} \end{aligned}$$

where $\{q^C\}$, $\{q^F\}$, and $\{q^T\}$ represent the displacement, $\{q_e^C\}$, $\{q_e^F\}$, and $\{q_e^T\}$ denote the nodal displacement, and $[N^C]$, $[N^F]$, and $[N^T]$ represent the matrix of shape functions for CBT, FSDT, and TSDT, respectively and given in Appendix A.

By substituting Eqs. (30)–(32) into Eqs. (20)–(23) and Eqs. (26)–(28), summing the energies for all elements, and then applying Eq. (29), we obtain the following equation of motion:

33 $$\begin{aligned} \left[ M\right] \ddot{\left\{ d_S\right\} }+\left[ K\right] \left\{ d_s\right\} = \left\{ 0\right\} , \end{aligned}$$

where $\left[ M\right]$ is the mass matrix and $\left[ K\right]$ is the stiffness matrix of the beam. The free vibration analysis is applied to Eq. (33) to calculate the natural frequencies by solving the eigenvalue problem Eq. (34), where $\Delta$ is the mode shape.

34 $$\begin{aligned} \left( \left[ K\right] -\omega ^2\left[ M\right] \right) \Delta = \left\{ 0\right\} \ \end{aligned}$$

### Finite element modeling using ANSYS

**Figure 4:**
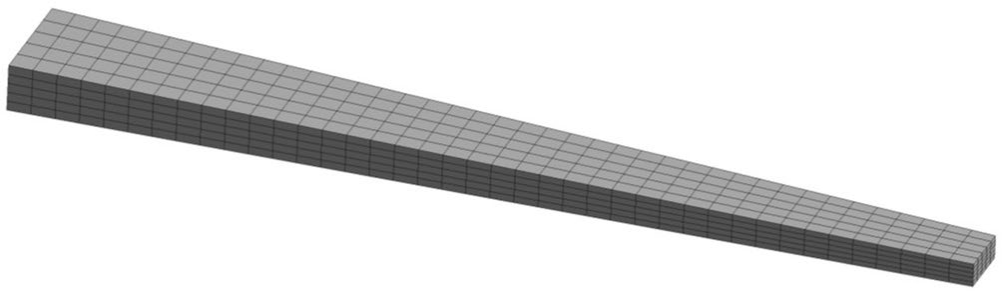
Discretization of a tapered beam model $(C_b$and$\ C_h=0.5)$.

To validate the obtained results from the developed mathematical models, 3-Dimensional Finite Element (3D-FE) simulations were conducted using ANSYS Workbench. A model of a tapered beam was created using the ANSYS Design Modeler, see Fig. 4. The simulations were performed for both isotropic and functionally graded material beam models. The geometrical parameters under investigation such as taper ratios $\left( C_b \ \text {and} \ C_h\right)$, aspect ratio $\left( \frac{L}{h_0}\right)$, and hub radius $\left( R\right)$ were integrated into the model as variables to easily control them through the Workbench’s parametric table. In addition to these parameters, other variables such as rotational speed $\left( {\bar{\Omega }}\right)$ and index parameters for the material distribution $\left( g_x \ \text {and} \ g_z\right)$ were also included to assess their impact on the natural frequencies of the beams.

Quadratic hexahedral SOLID185 elements were used to discretize the tapered beam geometry. Mesh controls were utilized to manipulate the mesh size of the beam edges, ensuring that only hexahedral elements were present in the mesh.

The implementation of functionally graded material properties in the model involved incorporating APDL commands within the modal module of Workbench. The material properties of the BFGM beam are determined at the coordinates of the centroid of each element, as described in Eq. (3). Thus, in order to achieve a uniform distribution of material properties, discretizing the beam into regular hexahedral elements is necessary. A typical mesh is shown in Fig. 4. The material properties were then assigned into the respective element.

### Uncertainties and stochastic modal analysis

As commonly recognized, system uncertainties encompassing material properties (*E*, *G*, and $\rho$) as well as material distribution ($g_x$ and $g_z$) in a rotating beam are subject to fluctuations in proximity to their designated values throughout processes such as measurement, structural element manufacturing, and structure assembly. Consequently, it is imperative to consider system parameters as stochastic rather than deterministic. Furthermore, the dynamic behavior of a rotating beam differs from that of a non-rotating beam due to the additional influence of centrifugal forces. The angular velocity ${\Omega }$ assumes a pivotal role in these phenomena and frequently exhibits variations in the vicinity of its operational speed. Thus, the angular velocity is inherently characterized by randomness. Henceforth, we will employ the notation $v_i$ ($i=1,2,\ldots ,6$) to represent the individual baseline random variables, with $v_1$ denoting ${\Omega }$, $v_i$ ($i=2,3,4$) representing the material properties *E*, *G*, and $\rho$, and $v_i$ ($i=5,6$) signifying the material gradient indices $g_x$ and $g_z$, respectively. These uncertainties have the potential to introduce variations in the components of the matrix [*K*] as outlined in Eq. (34). Given the uncertainty associated with the matrix [*K*], the natural frequencies themselves become stochastic variables. To ascertain the statistical characteristics of these natural frequencies, one can deduce them from the statistical properties of the baseline random parameters, employing a mean-centered second-order perturbation methodology.

The stochastic modal analysis of BFGM tapered rotating beams utilizes the mean-centered second moment method^41^. This perturbation approach is founded on the principle of expanding the random response around the mean values of the baseline random variables while retaining terms up to the second order. Within the framework of the mean-centered second-order method, the second-order approximate mean and the first-order approximated variance of natural frequencies can be mathematically expressed as follows:

35 $$\begin{aligned} E[\omega ]\cong \omega ^{(0)}+\frac{1}{2}\sum _{i=1}^{9}\sum _{i=1}^{9} \left (\omega _{,ii}^{(2)}+S_{v_i}^2 \right)\ \end{aligned}$$

36 $$\begin{aligned} {\textrm{Var}}[\omega ]\cong S_\omega ^2\approx \sum _{i=1}^{6} \left(w_i^{(1)} \right)^2S_{v_i}^2\ \end{aligned}$$

Here $\omega ^{(0)}$ represents the zero-order term of the natural frequency, equivalent to the deterministic natural frequency. $\omega _{,i}^{(1)}$ signifies the first-order term of the natural frequency concerning the random variable $v_i$, and $\omega _{,ii}^{(2)}$ corresponds to the second-order term of the natural frequency with respect to the random variable $v_i$. $S_\omega$ and $S_{v_i}$ are the standard deviation of $\omega$ and $v_i$, respectively. Hence the first and second-order term of natural frequency according to the finite difference approximation:

37 $$\begin{aligned} \omega _{,i}^{(1)}=\frac{\omega (v_i+\Delta v_i)-\omega (v_i)}{\Delta v_i}\ \end{aligned}$$

38 $$\begin{aligned} \omega _{,ii}^{(2)}=\frac{\omega (v_i+\Delta v_i)-2\omega (v_i)+\omega (v_i-\Delta v_i)}{(\Delta v_i)^2}\ \end{aligned}$$

where $\Delta v_i=v_i-\bar{v_i}$, with $\bar{v_i}$ denoting the mean value of the random variable $v_i$.

## Results and discussion

The axial and flap-wise bending vibration analysis of a rotating BFGM double-tapered cantilever beam is investigated for CBT, FSDT, and TSDT. The beam material varies according to the exponential law of distribution as given in Eq. (3). Several parameters such as the index parameters for the material distribution $(g_x$and$\ g_z)$, rotating speed $(\Omega )$, taper ratios $(C_b$and$\ C_h)$, hub radius (*R*), and aspect ratio $\left( \frac{L}{h_0}\right)$ are discussed. This section is divided into two subsections. The first subsection gives the convergence and validation of the present model with 3D-FE and the previously published literature. The second subsection presents and discusses the new findings. The dimensionless parameters used in the tables and figures are:

39 $$\begin{aligned} {\bar{\omega }}&= \omega \sqrt{\frac{\rho _0A_0L^4}{E_0I_0}} \nonumber \\ {\bar{\Omega }}&= \Omega \sqrt{\frac{\rho _0A_0L^4}{E_0I_0}} \nonumber \\ {\bar{R}}&= \frac{R}{L} \quad \end{aligned}$$

where ${\bar{\omega }}$ is the dimensionless natural frequency, ${\bar{\Omega }}$ is the dimensionless rotating speed, and ${\bar{R}}$ is the dimensionless hub radius.

To investigate the statistical characteristics of natural frequencies in BFGM rotating beams with varying baseline random variables, we consider the following scenarios:

**Case 1:** Only the rotational velocity ($\Omega$) is treated as a random variable.

**Case 2:** Only the material properties (*E*, *G*, and $\rho$) are considered as random variables.

**Case 3:** Only the material distribution indices ($g_x$ and $g_z$) are subject to randomness.

In order to obtain comparative results, we also employ the Monte Carlo (MC) method, generating 500 samples for analysis for case 1 only.

### Convergence and validation

The current model includes many parameters that should be taken into consideration to ensure a through verification procedure. These various parameters are the gradient index parameters for the material distribution $(g_x$and $\ g_z)$, taper ratios $(C_b$ and$\ C_h)$, rotating speed $({\bar{\Omega }})$, hub radius $({\bar{R}})$, and aspect ratio $\left( \frac{L}{h_0}\right)$.

#### Convergence rate

The convergence rates of the first five dimensionless natural frequencies are presented in Table 1 for CBT, FSDT, and TSDT and compared with 3D-FE results, where the subscripts f and ax denote flapping and axial modes respectively. The following data is used: $(b_0=0.04\ \text {m},\ h_0=0.02\ \text {m},$ $\rho _0=7850 {\text {kg/}}{\text {m}^3},\ E_0=210\ \text {GPa},\ \ \nu =0.3,\ \ \ \ C_b=C_h=0.5,\ g_x=g_x=0.4,\ {\bar{\Omega }}=2,$ and ${\bar{R}}=0.05)$ for both $\frac{L}{h_0}\ =20$and$\ 5$. The analysis of the data presented in Table 1 indicates a rapid convergence of results as the number of elements $(N_e)$ increases for the CBT, FSDT, and TSDT beam theories. Notably, when $N_e$ reaches 100, the accuracy of the obtained results becomes satisfactory for practical purposes, and hence this number of elements is employed for conducting free vibration analysis of rotating BFGM double-tapered beams. Remarkably, the outcomes derived from TSDT, FSDT, and 3D-FE analysis demonstrate a noteworthy level of agreement, particularly for thick beams at higher modes, when compared to the classical beam theory (CBT). This disparity can be attributed to the inherent limitations of CBT, which neglects the significant influence of shear deformation effects that become increasingly prominent in the higher modes of thick beam structures.

**Table 1:** Convergence of the first five dimensionless natural frequencies of a rotating BFGM double tapered beam.

$\frac{L}{h_0}$	Modes	Beam	$N_e$						3D-FE
		Theory	10	30	50	70	90	100	
20	${\bar{\omega }}_{1f}$	CBT	2.1773	2.1773	2.1773	2.1773	2.1773	2.1773	2.1793
		FSDT	2.1760	2.1760	2.1760	2.1760	2.1760	2.1760	
		TSDT	2.1763	2.1760	2.1760	2.1760	2.1760	2.1760	
	${\bar{\omega }}_{2f}$	CBT	4.3980	4.3976	4.3976	4.3976	4.3976	4.3976	4.3926
		FSDT	4.3841	4.3838	4.3838	4.3838	4.3838	4.3838	
		TSDT	4.4015	4.3850	4.3841	4.3840	4.3839	4.3839	
	${\bar{\omega }}_{1ax}$	CBT	6.9332	6.9312	6.9311	6.9311	6.9311	6.9311	6.8971
		FSDT	6.8825	6.8811	6.8810	6.8810	6.8810	6.8810	
		TSDT	6.9610	6.8863	6.8826	6.8819	6.8816	6.8816	
	${{\bar{\omega }}}_{3f}$	CBT	9.5265	9.5193	9.5191	9.519	9.519	9.5189	9.4232
		FSDT	9.4022	9.3979	9.3977	9.3976	9.3976	9.3976	
		TSDT	9.6134	9.4119	9.4020	9.4001	9.3994	9.3992	
	${{\bar{\omega }}}_{2ax}$	CBT	11.444	11.440	11.440	11.440	11.440	11.440	11.449
		FSDT	11.444	11.440	11.440	11.440	11.440	11.440	
		TSDT	11.444	11.440	11.440	11.440	11.440	11.440	
5	${\bar{\omega }}_{1f}$	CBT	2.1725	2.1724	2.1724	2.1724	2.1724	2.1724	2.1610
		FSDT	2.1519	2.1519	2.1519	2.1519	2.1519	2.1519	
		TSDT	2.1528	2.1524	2.1523	2.1523	2.1523	2.1523	
	${\bar{\omega }}_{2f}$	CBT	4.3486	4.3483	4.3483	4.3483	4.3483	4.3483	4.1888
		FSDT	4.1649	4.1647	4.1647	4.1647	4.1647	4.1647	
		TSDT	4.1779	4.1710	4.1702	4.1699	4.1698	4.1698	
	${\bar{\omega }}_{1ax}$	CBT	5.7218	5.7200	5.7198	5.7198	5.7198	5.7198	5.7195
		FSDT	5.7218	5.7199	5.7198	5.7198	5.7197	5.7197	
		TSDT	5.7218	5.7199	5.7198	5.7198	5.7197	5.7197	
	${{\bar{\omega }}}_{3f}$	CBT	6.7487	6.7469	6.7468	6.7468	6.7468	6.7468	6.2473
		FSDT	6.2075	6.2068	6.2067	6.2067	6.2067	6.2067	
		TSDT	6.2512	6.2288	6.2262	6.2253	6.2249	6.2248	
	${{\bar{\omega }}}_{4f}$	CBT	9.0759	9.0694	9.0691	9.0691	9.0690	9.0690	8.0921
		FSDT	8.0331	8.0315	8.0314	8.0314	8.0314	8.0314	
		TSDT	8.1239	8.0778	8.0727	8.071	8.0703	8.0701	

#### Effect of gradient index and aspect ratio

The provided data in Table 2 presents the first three dimensionless natural frequencies of a non-rotating $\left( \bar{{\Omega }}={0}\right)$ BFGM uniform beam $\left( {C_b}={C_h}={0}\right)$. The beam is analyzed using three different theories: TSDT, FSDT, and CBT. The frequencies obtained from these theories are compared with the reference values calculated using the dimensionless parameter ${\lambda }=\frac{\bar{{\omega }}}{\sqrt{{12}}}$ from^12^ using TSDT and 3D-FE results. The beam dimensions and material properties used in the analysis are $({b_0}=0.5\ \text{m},\ {h_0}=1\ \text{m},\ {\rho _0}=7850\ {\mathrm {kg/}}{\mathrm {m^3}}$, $\ {E_0}=210\ \text{GPa},\ \ {\nu }=0.3,$ and ${\bar{R}}=0)$. Two different aspect ratios, $\frac{L}{h_0} = 20$ and 5, are considered.

It can be observed from Table 2 that TSDT, FSDT, and 3D-FE demonstrate a commendable level of agreement with the reference results, regardless of whether the beams are thick or thin. This indicates the robustness and reliability of these methods in accurately predicting the natural frequencies. While CBT shows good agreement with the reference results for the 1st mode of a thin beam $\left( \frac{L}{h_0}=20\right)$, its performance deviates from the TSDT, FSDT, 3D-FE, and^12^ results for other modes and thicker beams. However, it is worth noting that for the third mode of $\left( \frac{L}{h_0}=5\right)$, which corresponds to the first axial mode, all three theories (TSDT, FSDT, and CBT) exhibit satisfactory agreement with both the 3D-FE and reference results.

Furthermore, the results show that as the gradient index $\left( {g_x}=\ {g_z}\right)$ increases, the frequency values tend to decrease for all three modes across all methods, and both $\frac{L}{h_0}=20\ \text{and}\ 5$.

**Table 2:** The first three dimensionless natural frequencies of a non-rotating BFGM uniform beam.

$\lambda$	$g_x=g_z$	$\frac{L}{h_0}=20$					$\frac{L}{h_0}=5$				
		TSDT	FSDT	CBT	Ref.^12^	3D_FE	TSDT	FSDT	CBT	Ref.^12^	3D_FE
$\lambda _1$	0	1.0130	1.0130	1.0145	1.0130	1.0147	0.9847	0.9843	1.0072	0.9848	0.9911
	0.2	0.9515	0.9515	0.9529	0.9515	0.9533	0.9261	0.9257	0.9463	0.9261	0.9324
	0.6	0.8329	0.8329	0.8340	0.8329	0.8346	0.8125	0.8123	0.8288	0.8126	0.8187
	1	0.7214	0.7214	0.7223	0.7214	0.7230	0.7055	0.7054	0.7182	0.7055	0.7113
$\lambda _2$	0	6.2750	6.2742	6.3394	6.2758	6.2867	5.3237	5.3011	6.0419	5.3263	5.3491
	0.2	6.1516	6.1508	6.2148	6.1524	6.1634	5.2168	5.1944	5.9229	5.2195	5.2418
	0.6	5.8762	5.8758	5.9365	5.8771	5.8879	4.9832	4.9635	5.6596	4.9861	5.0060
	1	5.5687	5.5688	5.6254	5.5696	5.5801	4.7272	4.7127	5.3675	4.7302	4.7463
$\lambda _3$	0	17.2569	17.2513	17.6687	17.2627	17.2930	7.8541	7.8541	7.8541	7.8540	7.8883
	0.2	17.1308	17.1254	17.5391	17.1368	17.1671	7.5388	7.5388	7.5388	7.5387	7.5738
	0.6	16.7945	16.7911	17.1911	16.8007	16.8300	6.9271	6.9271	6.9271	6.9270	6.9633
	1	16.3556	16.3561	16.7349	16.3618	16.3889	6.3415	6.3415	6.3415	6.3414	6.3783

Note that $$\lambda = {\bar{\omega }}/\sqrt{12}$$.

#### Effect of rotating speed and hub radius

Table 3 presents the first three dimensionless natural frequencies of a rotating double-tapered homogeneous $(g_x=g_z=0)$ beam analyzed using three different theories: TSDT, FSDT, and CBT. The frequencies obtained from these theories are compared with the reference values from^23^ using FSDT and 3D-FE results. The analysis is conducted with two different hub radii $(R=0 \ \& \ L)$, and three rotating speeds $(\Omega =0,\ 50 \text{ and 100 rad/s})$. The beam dimensions and material properties used in the analysis are $(b_0=0.05 \text { m},\ h_0=0.01 \text { m},\ L=2 \text { m},\ C_h=C_b=0.5,\ \rho _0=7800 {\text {kg/}}{\text {m}^3},\ E_0=214 \text { GPa},\ G_0=82.2 \text { GPa},\ \text {and } \nu =0.3)$. The comparison of the results presented in Table 3 reveal a notable level of agreement among the TSDT, FSDT, CBT, and 3D-FE methods with the reference results. Particularly, the CBT method exhibits excellent agreement in this specific example, where the beam under investigation is characterized by being extremely thin.

Regarding the effect of rotating speed, it is evident that an increase in rotational velocity leads to an increase in the natural frequencies. Furthermore, it can be inferred that the eigenfrequencies exhibit an increasing trend with the rise in angular velocity and hub radius. This behavior can be attributed to the centrifugal stiffness effect. As the angular velocity and hub radius increase, the centrifugal force acting on the beam also increases, resulting in a higher stiffness. Consequently, the eigenfrequencies of the beam are influenced and exhibit an upward trend in response.

**Table 3:** The first three natural frequencies of a homogenous rotating double tapered for various rotating speed, and hub radii.

${\omega}_i$	$\Omega$	$R=0$					$R=L$				
(Hz)	(rad/s)	TSDT	FSDT	CBT	Ref.^23^	3D-FE	TSDT	FSDT	CBT	Ref.^23^	3D-FE
${\omega}_1$	0	2.7826	2.7826	2.7826	2.7826	2.7856	2.7826	2.7826	2.7826	2.7826	2.7856
	50	9.0874	9.0873	9.0874	9.0872	9.0929	13.702	13.702	13.702	13.702	13.708
	100	17.004	17.004	17.005	17.004	17.014	26.581	26.580	26.581	26.581	26.590
${\omega}_2$	0	11.760	11.759	11.760	11.759	11.774	11.760	11.759	11.760	11.759	11.774
	50	21.585	21.584	21.585	21.584	21.600	29.958	29.957	29.958	29.957	29.974
	100	37.673	37.672	37.674	37.672	37.695	55.820	55.817	55.820	55.819	55.841
${\omega}_3$	0	29.228	29.220	29.225	29.222	29.261	29.228	29.220	29.225	29.222	29.261
	50	40.499	40.493	40.497	40.494	40.532	51.999	51.994	51.998	51.995	52.031
	100	62.829	62.823	62.828	62.824	62.867	89.785	89.778	89.785	89.782	89.821

### New results

This subsection presents the vibrational behavior of a BFGM double tapered rotating beam. The beam material properties are $E_0=210$ GPa, $\rho _0=7850 {\text {kg/}}{\text {m}^3}$, and $\nu =0.3$, and the beam dimensions are $b_0=0.04$ m and $L=0.4$ m. Several parameters such as the index parameters ($g_x$ and $g_z$) for the material distribution, rotating speed ($\Omega$), hub radius (*R*), and aspect ratio ($\frac{L}{h_0}$) are considered.

#### Effect of aspect ratio on natural frequencies: CBT, FSDT, and TSDT comparison

**Figure 5:**
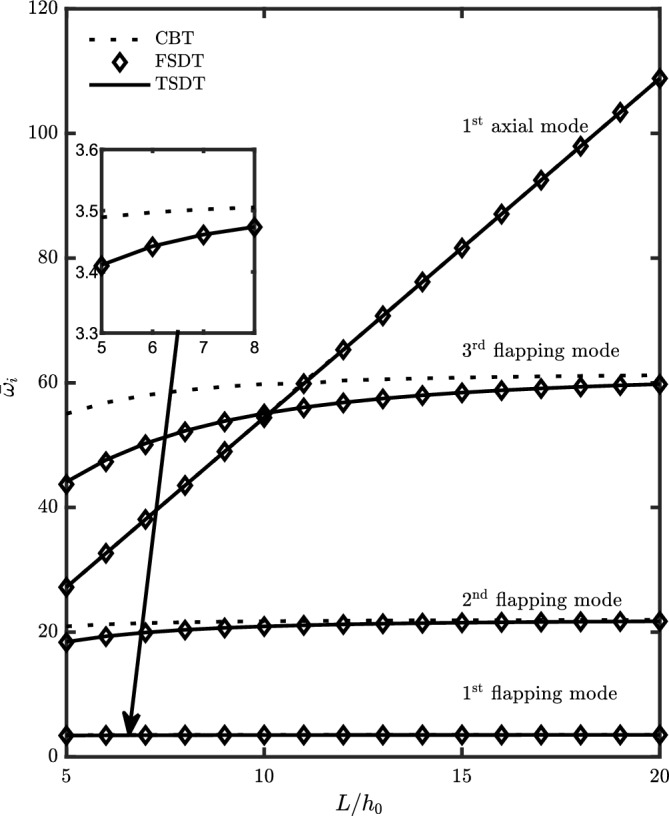
The first four dimensionless natural frequencies versus aspect ratio.

Figure 5 presents the variation of the first four dimensionless natural frequencies of a non-rotating $\left( \Omega =0 {\text { rad/}}{\text {s}}\right)$ homogeneous $\left( g_x=g_z=0\right)$ uniform $\left( C_b=C_h=0\right)$ beam versus the aspect ratio. The results demonstrate a correlation between the aspect ratio and the natural frequency, indicating that an increase in the aspect ratio leads to a higher natural frequency. However, this increase becomes less significant for the flapping modes as the aspect ratio reaches higher values. Furthermore, there is a deviation between the CBT theory and the other two theories, FSDT and TSDT, which is more pronounced for higher modes of flapping vibration. However, in the case of axial vibration, there is a good agreement between the three theories. Additionally, there is a good agreement between the FSDT and TSDT theories, particularly for low aspect ratios or thick beams.

Moreover, with changing aspect ratio, the axial and flapping modes may interchange their order. This means that as the aspect ratio varies, there is a possibility for the axial mode and the flapping mode to switch positions in the mode sequence, resulting in a change in their relative order.

#### Effect of taper ratio on natural frequencies: CBT, FSDT, and TSDT comparison

**Figure 6:**
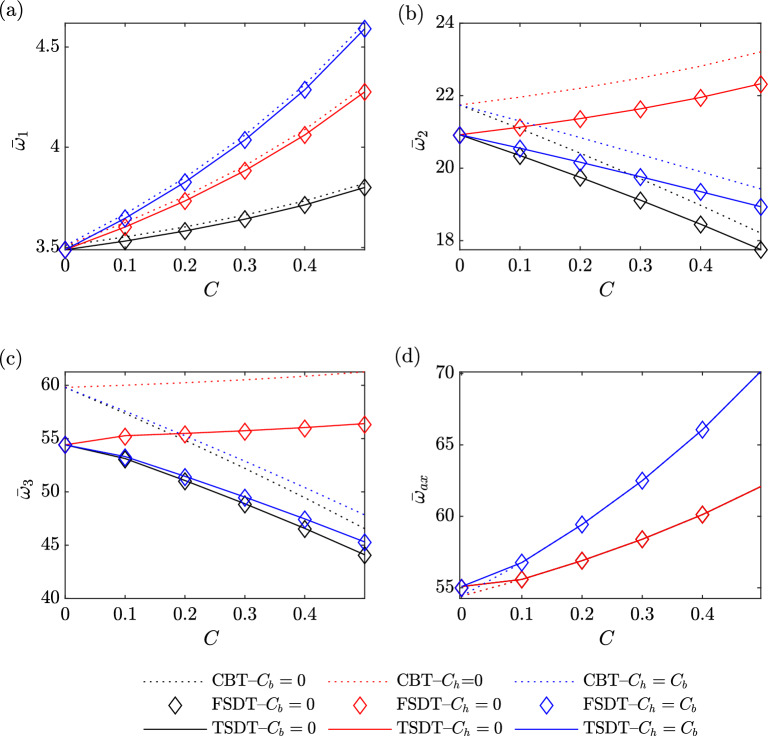
The first four dimensionless natural frequencies of a non-rotating homogeneous beam versus taper ratios.

Figure 6 presents the variation of the first four dimensionless natural frequencies of a non-rotating $\left( \Omega =0\ {\text {rad/}}{\text {s}}\right)$ homogeneous $\left( g_x=g_z=0\right)$ beam versus taper ratios for aspect ratio $\left( \frac{L}{h_0}=10\right)$. Subfigures (a) and (d) depict the first flapping and axial modes, respectively. These subfigures provide clear evidence of a direct relationship between the taper ratio and the natural frequency, indicating that an increase in the taper ratio leads to an increase in the natural frequency. Furthermore, the highest natural frequencies are achieved when the taper ratio is increased in both the height and width directions, followed by tapering in the width direction only and then tapering in the height direction only. Notably, when the taper ratio is changed in either the height or width direction alone, the first axial mode exhibits identical natural frequencies due to the beam’s consistent width and height. Moreover, a slight deviation can be observed in the results obtained from the CBT method compared to both the FSDT and TSDT methods in the first flapping mode.

In Subfigures (b) and (c), the second and third flapping modes are presented, respectively. These subfigures clearly demonstrate that an increase in the taper ratio in the width direction corresponds to an increase in the natural frequency. Conversely, increasing the taper ratio in both the height and width directions or in the height direction alone leads to a decrease in the natural frequency. Furthermore, the highest natural frequencies are obtained when the taper ratio is increased in the width direction only, followed by tapering in both the height and width directions and then tapering in the height direction only. Additionally, the observed deviations in the results obtained from the CBT method, compared to both the FSDT and TSDT methods, are more pronounced in the third mode than in the second mode.

#### Effect of rotating speed and hub radius on natural frequencies: CBT, FSDT, and TSDT comparison

**Figure 7:**
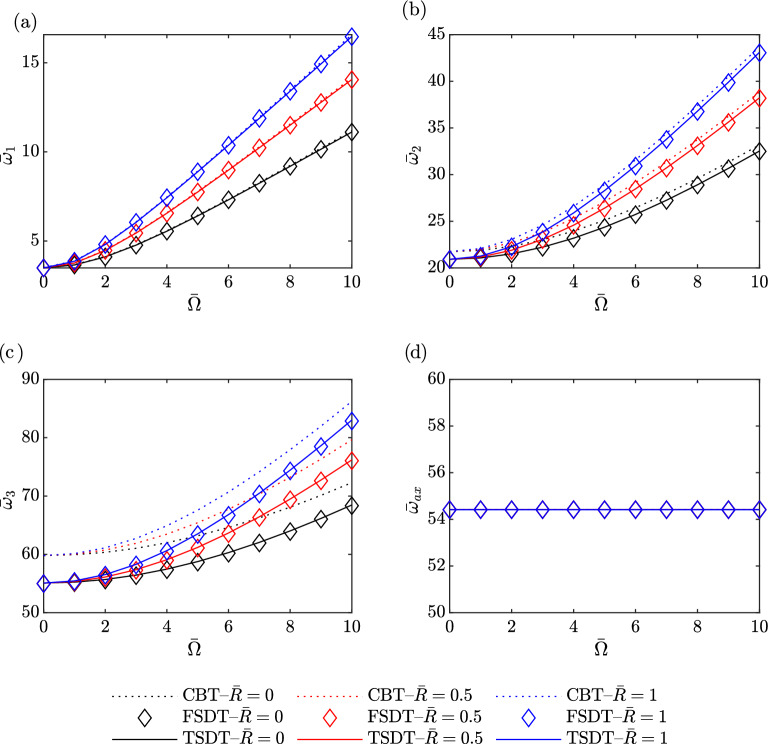
The first four dimensionless natural frequencies of a homogeneous uniform beam versus rotating speed and hub radius.

Figure 7 presents the variation of the first four dimensionless natural frequencies of a homogeneous $(g_x=g_z=0)$ uniform $(C_b=C_h=0)$ beam versus rotating speed and hub radius for aspect ratio $\left( \frac{L}{h_0}=10\right)$. Figure 7a–c show the first three flapping modes, respectively. Increasing the rotating speed is associated with higher natural frequencies in all three flapping modes, attributed to the stiffening effect of centrifugal force. The hub radius also influences the natural frequency, particularly at higher rotating speeds, where the larger hub radius induces a stronger centrifugal force. Both increasing rotating speed and larger hub radius contribute to higher natural frequencies due to the enhanced effect of centrifugal force as shown in Eq. (24). Furthermore, the deviation between the CBT method and the FSDT and TSDT methods increases with higher modes, indicating that differences in predictions become more pronounced as the mode number increases.

Figure 7d indicates that the rotating speed and hub radius do not have a significant effect on the natural frequency in the first axial mode.

#### Effect of gradient index on natural frequencies: TSDT

**Figure 8:**
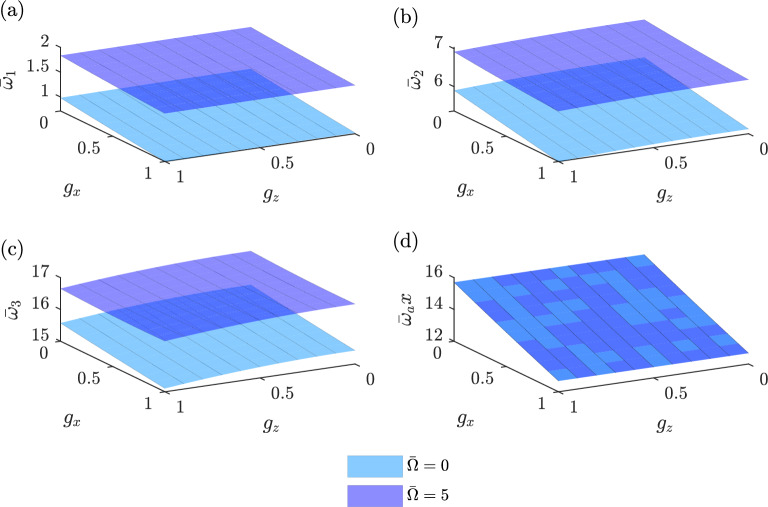
Comparison of the first four dimensionless natural frequencies between non-rotating and rotating uniform beams versus the gradient index.

The findings presented in Fig. 8 demonstrate the relationship between the gradient indexes $g_x$ and $g_z$ and the first four dimensionless natural frequencies of a uniform beam for two dimensionless speeds $\left( {\bar{\Omega }}=0\ \text {and}\ 5\right)$ according to TSDT. Figure 8a–c results indicate a clear inverse correlation, where the dimensionless natural frequencies decrease as the gradient index increases. Furthermore, it is observed that the gradient index in the *x*-direction ($g_x$) has a more significant impact on the natural frequencies compared to the gradient index in the *z*-direction ($g_z$). The natural frequencies of all three flapping modes exhibit an increase as the rotating speed is increased. Figure 8d illustrates that the natural frequency in the first axial mode remains largely unaffected by changes in the rotating speed.

#### Uncertainties and stochastic results

**Figure 9:**
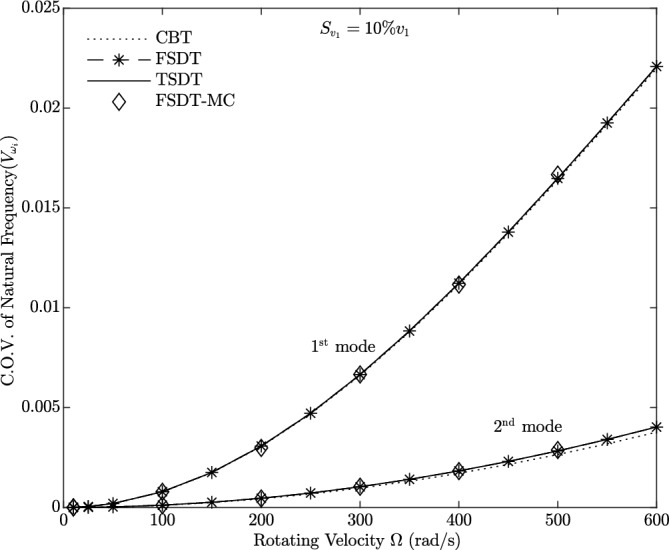
C.O.V. of 1st and 2nd natural frequencies for random rotating velocity (Case 1) versus rotating velocity for three beam theories using the second-order perturbation method compared to Monte Carlo (MC).

**Figure 10:**
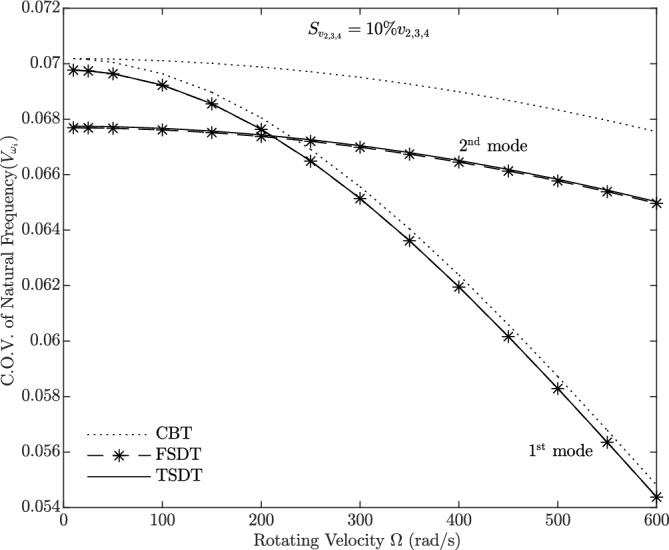
C.O.V. of 1st and 2nd natural frequencies for random material properties (Case 2) versus rotating velocity for three beam theories using the second-order perturbation method.

**Figure 11:**
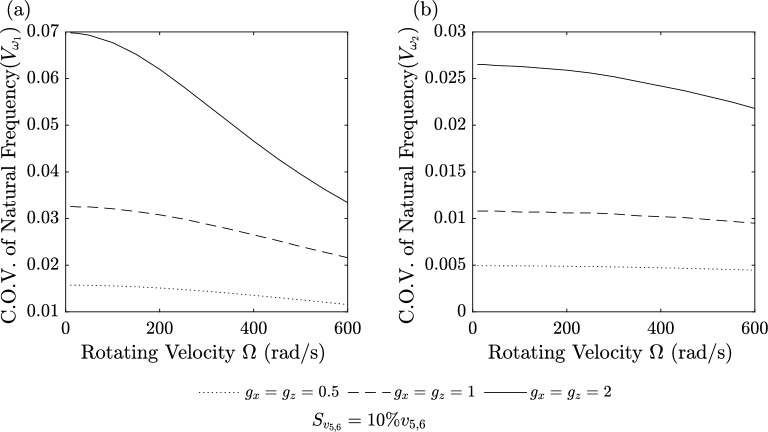
C.O.V. for random material distribution (Case 3) versus rotating velocity at $g_x=g_z=[0.5\ 1\ 2]$ for TSDT in second-order perturbation method, (**a**) 1st mode and (**b**) 2nd mode.

**Figure 12:**
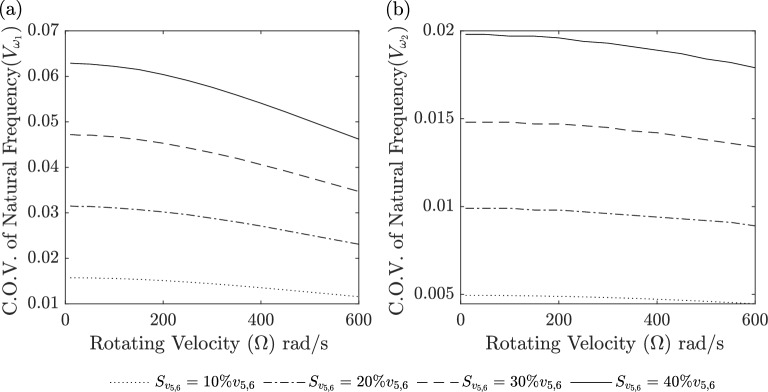
C.O.V. for random material distribution (Case 3) versus rotating velocity at $g_x=g_z=0.5$ for TSDT in second-order perturbation method, (**a**) 1st mode and (**b**) 2nd mode.

Figures 9, 10, 11 and 12 show the Coefficient of Variation (C.O.V.) for the 1st and 2nd natural frequencies, denoted as $V_{\omega _i}$ $(i=1,2)$, of a uniform beam with ${\bar{R}}=0.1$ and $\frac{L}{h_0}=10$. These plots are generated as a function of rotational velocity. Figure 9 illustrates the Coefficient of Variation (C.O.V.) for the case where only the rotational velocity is treated as a random variable (Case 1), with a standard deviation of 10% $\Omega$. The analysis is conducted on a homogeneous beam ($g_x=g_z=0$) using the three different theories. Strong agreement is observed between the second-order perturbation and Monte Carlo methods, confirming the accuracy of the results across all rotational speeds. It is evident that $V_{\omega _i}$ increases with an increase in rotational velocity. Consequently, the C.O.V. of the first mode natural frequency remains nearly identical across all three theories, with only a slight deviation observed in the second mode, particularly between CBT and the other two theories, FSDT and TSDT.

Figure 10 represents the Coefficient of Variation (C.O.V.) for the case where only the material properties are considered as random variables (Case 2), with a standard deviation of 10% for *E*, *G*, and $\rho$. The analysis is performed on a homogeneous beam ($g_x=g_z=0$) using the three theories. It is observed that $V_{\omega _i}$ decreases with an increase in rotational velocity. Consequently, the C.O.V. of the natural frequency in the case of CBT deviates from that of FSDT and TSDT, with this deviation increasing from the first to the second mode. A comparison between Figures 9 and 10 reveals that the randomness of material properties has a more significant influence on $V_{\omega _i}$ than the randomness of rotational velocity within the speed range of 0 to 600 rad/s.

Figures 11 and 12 represent the Coefficient of Variation (C.O.V.) for the case where only the material distribution is subject to randomness (Case 3) according to TSDT. Figure 11a and b is applied to a BFGM beam ($g_x=g_z=[0.5\ 1\ 2]$) with a standard deviation of 10% for $g_x$ and $g_z$. It is observed that $V_{\omega _i}$ decreases as the rotational velocity increases. Additionally, $V_{\omega _i}$ exhibits higher values for larger gradient indices. Figure 12a and b is applied to a BFGM beam ($g_x=g_z=0.5$) with standard deviations of [10 20 30 40 ]% for $g_x$ and $g_z$. It is observed that $V_{\omega _i}$ decreases as the rotational velocity increases. Additionally, $V_{\omega _i}$ exhibits higher values for larger standard deviations.

## Conclusions

In this study, a comparative analysis of free vibration behavior and stochastic analysis was conducted for rotating double-tapered beams composed of BFGM using three different beam theories: CBT, FSDT, TSDT. The material properties of the beams were characterized by an exponential distribution model. The investigation aimed to assess the impact of various parameters such as material distribution, taper ratios, aspect ratio, hub radius, and rotational speed on the natural frequencies. It also focused on studying uncertainties in material properties, material distribution, and rotational velocity. The results obtained from the study demonstrated the convergence and validation of the proposed model by comparing it with 3D-FE simulations conducted using ANSYS and previously published research. The convergence analysis indicated that the accuracy of the results reached a satisfactory level for practical purposes. Additionally, the stochastic analysis outcomes, derived using the mean-centered second-order perturbation approach, underwent validation through comparison with the Monte Carlo method. Furthermore, the comparison between the beam theories showed that FSDT and TSDT exhibited a greater agreement with the 3D-FE simulations, particularly for thick beams, compared to CBT. This finding highlights the importance of considering shear deformation effects, which become more significant in thick beam structures. The results of the study showed that the natural frequencies of the rotating beams increased with increasing taper ratio in the width direction, aspect ratio, rotational speed, and hub radius. The results also showed that the natural frequencies of the beams decreased with increasing gradient index. In the context of random parameters, distinct trends emerge in the coefficient of variation (C.O.V.) concerning rotational velocity and various sources of uncertainty. Specifically, an increase in rotational velocity results in an increasing C.O.V. for random velocity, while random material properties and material distribution exhibit a decreasing C.O.V. trend with increasing velocity. Notably, for the first mode, a consistent C.O.V. is observed across all three theories for random rotational velocity. However, in the case of random material properties, discernible deviations are noted for CBT compared to FSDT and TSDT.

## Data Availability

The datasets generated during and/or analyzed during the current study are available from the corresponding author on reasonable request.

## Appendix A: displacement, nodal displacement and matrix of shape functions

In this section, a comprehensive overview of the details pertaining to displacement, nodal displacement, and the matrix of shape functions is presented. The displacements are

40 $$\begin{aligned} \begin{aligned} \{q^C\} =&\begin{bmatrix} u_0^C(x,t) \ \ \ w_0^C(x,t) \end{bmatrix}^\intercal ,\\ \{q^F\} =&\begin{bmatrix} u_0^F(x,t) \ \ \ w_0^F(x,t) \ \ \ \phi ^F(x,t) \end{bmatrix}^\intercal ,\\ \{q^T\} =&\begin{bmatrix} u_0^T(x,t) \ \ \ w_0^T(x,t) \ \ \ \psi ^T(x,t) \end{bmatrix}^\intercal , \end{aligned} \end{aligned}$$

the vectors of nodal displacements are

41 $$\begin{aligned} \begin{aligned} \{q_e^C\} =&\begin{bmatrix} U_1(t) \ \ W_1(t) \ \ W_1^\prime (t) \ \ U_2(t) \ \ W_2(t) \ \ W_2^\prime (t) \end{bmatrix}^\intercal \\ \{q_e^F\} =&\begin{bmatrix} U_1(t) \ \ W_1(t) \ \ \phi _1(t) \ \ W_1^\prime (t) \ \ \phi _1^\prime (t) \ \ U_2(t) \ \ W_2(t) \ \ \phi _2(t) \ \ W_2^\prime (t) \ \ \phi _2^\prime (t) \end{bmatrix}^\intercal \\ \{q_e^T\} =&\begin{bmatrix} U_1(t) \ \ W_1(t) \ \ W_1^\prime (t) \ \ \psi _1(t) \ \ U_2(t) \ \ W_2(t) \ \ W_2^\prime (t) \ \ \psi _2(t) \end{bmatrix}^\intercal , \end{aligned} \end{aligned}$$

and the shape functions are

42 $$\begin{aligned} &[N^C] = \begin{bmatrix} 1-\frac{x}{\ell } &{} 0 &{} 0 &{} \frac{x}{\ell } &{} 0 &{} 0 \\ 0 &{} 1-3\left( \frac{x}{\ell }\right) ^2+2\left( \frac{x}{\ell }\right) ^3 &{} x\left( 1-\frac{x}{\ell }\right) ^2 &{} 0 &{} 3\left( \frac{x}{\ell }\right) ^2-2\left( \frac{x}{\ell }\right) ^3 &{} x\left( \left( \frac{x}{\ell }\right) ^2-\frac{x}{\ell }\right) \end{bmatrix},\\&[N^F] = \begin{bmatrix} 1-\frac{x}{\ell } &{} 0 &{} 0 &{} 0 &{} 0 &{} \frac{x}{\ell } &{} 0 &{} 0 &{} 0 &{} 0 \\ 0 &{} 1-3\left( \frac{x}{\ell }\right) ^2+2\left( \frac{x}{\ell }\right) ^3 &{} 0 &{} x\left( 1-\frac{x}{\ell }\right) ^2 &{} 0 &{} 0 &{} 3\left( \frac{x}{\ell }\right) ^2-2\left( \frac{x}{\ell }\right) ^3 &{} 0 &{} x\left( \left( \frac{x}{\ell }\right) ^2-\frac{x}{\ell }\right) &{} 0 \\ 0 &{} 0 &{} 1-3\left( \frac{x}{\ell }\right) ^2+2\left( \frac{x}{\ell }\right) ^3 &{} 0 &{} x\left( 1-\frac{x}{\ell }\right) ^2 &{} 0 &{} 0 &{} 3\left( \frac{x}{\ell }\right) ^2-2\left( \frac{x}{\ell }\right) ^3 &{} 0 &{} x\left( \left( \frac{x}{\ell }\right) ^2-\frac{x}{\ell }\right) \end{bmatrix},\\&[N^T] = \begin{bmatrix} 1-\frac{x}{\ell } &{} 0 &{} 0 &{} 0 &{} \frac{x}{\ell } &{} 0 &{} 0 &{} 0 \\ 0 &{} 1-3\left( \frac{x}{\ell }\right) ^2+2\left( \frac{x}{\ell }\right) ^3 &{} x\left( 1-\frac{x}{\ell }\right) ^2 &{} 0 &{} 0 &{} 3\left( \frac{x}{\ell }\right) ^2-2\left( \frac{x}{\ell }\right) ^3 &{} x\left( \left( \frac{x}{\ell }\right) ^2-\frac{x}{\ell }\right) &{} 0 \\ 0 &{} 0 &{} 0 &{} 1-\frac{x}{\ell } &{} 0 &{} 0 &{} 0 &{} \frac{x}{\ell } \end{bmatrix} \end{aligned}$$

where $\xi =\frac{x}{\ell }$, $U_1$ and $U_2$ are the axial displacements, $W_1$ and $W_2$ are the transverse displacements, $W_1^\prime$ and $W_2^\prime$ are the pure bending slopes of CBT, $\Phi _1$ and $\Phi _2$ are the cross-section rotations of FSDT, and $\Psi _1$ and $\Psi _2$ are the deformed line slopes at $z=0$ of TSDT at nodes 1 and 2, respectively.

## List of symbols

*A*(*X*): Beam cross-sectional area as a function of *X* (m^2^)
$A_0$: Beam cross-sectional area at the beam root (m^2^)
*b*(*X*): Beam width as a function of *X* (m)
$b_0$: Beam width at the beam root (m)
$b_L$: Beam width at the beam free end (m)
$C_b$: Width taper ratio
$C_h$: Thickness taper ratio
C.O.V: Coefficient of variation
$$\{d_s\}$$: Displacement vector
*E*: Young’s modulus (Pa)
*E*[*omega*]: Mean approximated natural frequencies
$g_x$: Gradient index through the longitudinal direction
$g_z$: Gradient index through the thickness direction
*h*(*X*): Beam thickness or height as a function of *X* (m)
$h_0$: Beam thickness at the beam root (m)
$h_L$: Beam thickness at the beam free end (m)
$I_0$: Beam second area moment of inertia at the beam root (m$^4$)
[*K*]: Stiffness matrix
KE: Kinetic energy
*L*: Beam total length (m)
$L_i$: Offset distance from the origin to the $i^{th}$ element
$$\ell$$: Element length (m)
[*M*]: Mass matrix
MC: Monte Carlo
$N_e$: Number of elements
*P*(*x*, *z*): Beam effective material properties
$PE_{cf}$: Potential energy due to centrifugal force
$PE_s$: Potential energy due to stress field
*R*: Hub radius (*m*)
${\bar{R}}$: Dimensionless hub radius (*R*/*L*)
$S_\omega$: Standard deviation of natural frequency
$S_{v_i}$: Standard deviation of random variables
*t*: Time (*s*)
*u*(*x*, *z*, *t*): Axial displacement field
$u_0$: Axial displacement on the neutral axis (i.e., $z=0$)
$U_1$, $U_2$: Axial displacements at nodes 1 and 2 respectively
$$v_i$$: Random variables
$\bar{v_i}$: Mean value of the random variable
$${\mathbb {V}}$$: Volume (m^3^)
$Var{[}\omega {]}$: First-order approximated variance of natural frequencies
*w*(*x*, *z*, *t*): Lateral or flapping displacement field
$w_0$: Lateral displacement on the neutral axis (i.e., $z=0$)
$\frac{\partial w_0}{\partial x}$: Pure bending cross-sectional slope in CBT
$W_1$, $W_2$: Lateral displacements at nodes 1 and 2 respectively
$W_1^\prime$, $W_2^\prime$: Pure bending slopes of CBT at nodes 1 and 2 respectively
*x*, *y*, *z*: Local coordinates
*X*, *Y*, *Z*: Global coordinates
$${\mathbb {Z}}$$: Rotating axis
$\gamma _{xz}$: Shear strain field
$$\varepsilon$$: Strain vector
$\varepsilon _{xx}$: Axial strain field
$\lambda ={\bar{\omega }}/\sqrt{12}$: Another dimensionless natural frequency form
$$\nu$$: Poisson’s ratio
$$\rho$$: Density ($$\text {kg/m}^3$$)
$$\sigma$$: Stress vector
$$\sigma _{xx}$$: Axial stress
$$\tau _{xz}$$: Shear stress
$\phi$: Cross-section rotation in FSDT
$\Phi _1$, $\Phi _2$: Cross-section rotations of FSDT at nodes 1 and 2 respectively
$\psi$: Cross-section slope at $z=0$ in TSDT
$\Psi _1$, $\Psi _2$: Line slopes at $z=0$ of TSDT at nodes 1 and 2 respectively
$\omega$: Natural frequency (rad/s)
$\omega ^{(0)}$: Zero-order term of natural frequency
$\omega _{,i}^{(1)}$: First-order term of natural frequency
$\omega _{,ii}^{(2)}$: Second-order term of natural frequency
${\bar{\omega }}$: Dimensionless natural frequency($\omega \sqrt{\rho _0A_0L^4/E_0I_0}$)
$\Omega$: Rotating velocity (rad/s)
${\bar{\Omega }}$: Dimensionless rotating velocity($\Omega \sqrt{\rho _0A_0L^4/E_0I_0}$)
$$\delta$$: Virtual operator
$\Delta$: Mode shape vector
